# Elevated transcriptional levels of aldolase A (ALDOA) associates with cell cycle-related genes in patients with NSCLC and several solid tumors

**DOI:** 10.1186/s13040-016-0122-4

**Published:** 2017-02-07

**Authors:** Fan Zhang, Jie-Diao Lin, Xiao-Yu Zuo, Yi-Xuan Zhuang, Chao-Qun Hong, Guo-Jun Zhang, Xiao-Jiang Cui, Yu-Kun Cui

**Affiliations:** 1 Guangdong Provincial Key Laboratory for Breast Cancer Diagnosis and Treatment, Cancer Hospital of Shantou University Medical College, Shantou, 515041 China; 2 Sun Yat-Sen University Cancer Center, State Key Laboratory of Oncology in South China, Collaborative Innovation Center for Cancer Medicine, Guangzhou, 510060 China; 3 Department of Surgery, Samuel Oschin Comprehensive Cancer Institute, Cedars-Sinai Medical Center, Los Angeles, CA 90048 USA

**Keywords:** ALDOA, Transcripts, Cell cycle, Cancer

## Abstract

**Background:**

Aldolase A (ALDOA) is one of the glycolytic enzymes primarily found in the developing embryo and adult muscle. Recently, a new role of ALDOA in several cancers has been proposed. However, the underlying mechanism remains obscure and inconsistent. In this study, we tried to investigate ALDOA-associated (AA) genes using available microarray datasets to help elucidating the role of ALDOA in cancer.

**Results:**

In the dataset of patients with non-small-cell lung cancer (NSCLC, E-GEOD-19188), 3448 differentially expressed genes (DEGs) including ALDOA were identified, in which 710 AA genes were found to be positively associated with ALDOA. Then according to correlation coefficients between each pair of AA genes, ALDOA-associated gene co-expression network (GCN) was constructed including 182 nodes and 1619 edges. 11 clusters out of GCN were detected by ClusterOne plugin in Cytoscape, and only 3 of them have more than three nodes. These three clusters were functionally enriched. A great number of genes (43/79, 54.4%) in the biggest cluster (Cluster 1) primarily involved in biological process like cell cycle process (*P*_a_ = 6.76E-26), mitotic cell cycle (*P*_a_ = 4.09E-19), DNA repair (*P*_a_ = 1.13E-04), M phase of meiotic cell cycle (*P*_a_ = 0.006), positive regulation of ubiquitin-protein ligase activity during mitotic cell cycle (*P*_a_ = 0.014). AA genes with highest degree and betweenness were considered as hub genes of GCN, namely CDC20, MELK, PTTG1, CCNB2, CDC45, CCNB1, TK1 and PSMB2, which could distinguish cancer from normal controls with ALDOA. Their positive association with ALDOA remained after removing the effect of HK2 and PKM, the two rate limiting enzymes in glycolysis. Further, knocking down ALDOA blocked breast cancer cells in the G0/G1 phase under minimized glycolysis. All suggested that ALDOA might affect cell cycle progression independent of glycolysis. RT-qPCR detection confirmed the relationship of ALDOA with CDC45 and CCNB2 in breast tumors. High expression of the hub genes indicated poor outcome in NSCLC. ALDOA could improve their predictive power.

**Conclusions:**

ALDOA could contribute to the progress of cancer, at least partially through its association with genes relevant to cell cycle independent of glycolysis. AA genes plus ALDOA represent a potential new signature for development and prognosis in several cancers.

## Background

In mammalian tissues, three aldolase isozymes (A, B and C), encoded by three different genes, are differentially expressed during development. Aldolase A (ALDOA), also known as fructose-bisphosphate aldolase A, is one of the glycolytic enzymes that catalyze the reversible conversion of fructose-1, 6-bisphosphate to glyceraldehyde-3-phosphate and dihydroxyacetone phosphate. ALDOA is primarily found in the developing embryo and adult muscle, and contributes to various cellular functions and biological process related to muscle maintenance, regulation of cell shape and mobility, striated muscle contraction, actin filament organization and ATP biosynthetic process. ALDOA deficiency probably results in myopathy and hemolytic anemia [1–3].

Recently, a new role of ALDOA has been proposed, given that ALDOA is highly expressed in a variety of malignant cancers, including human lung cancer [4], osteosarcoma [5], colorectal cancer [6], oral squamous cell carcinomas [7] and hepatocellular carcinomas [8]. It could serve as a diagnostic and prognostic marker. Although elevated ALDOA level has been observed in these tumors, the underlying mechanism remains obscure and inconsistent. Some assumed that since glycolysis in rapidly growing tumor cells was up to 200 times faster than those of their normal tissues (Warburg effect), ALDOA expression would be also increased as an enzyme of this process [5]. However, others demonstrated that ALDOA probably played a non-metabolic role to facilitate cell proliferation [9]. Although RNA interference of ALDOA has been shown to inhibit cell proliferation in Ras-transformed NIH-3 T3 cells, there was no report on the potential mechanism or associated genes relevant to the role of ALDOA played in cell proliferation [10].

In the glycolysis pathway, ALDOA functions at the fourth step, followed by glyceraldehyde-3-phosphate dehydrogenase (GAPDH), another glycolytic enzyme at the sixth step. GAPDH is regarded as a housekeeping gene, but its expression is not always constant, especially in cancer, and has been supposed to correlate with cell cycle-dependent genes [11]. However, little was known about the associated genes of ALDOA and its mechanism other than glycolysis, albeit its potential role in tumors. In this study, we tried to investigate ALDOA-associated genes (AA genes) with the utilization of publicly available microarray datasets, which would help elucidating the role for ALDOA played in cancer. Since the effect of ALDOA on lung cancer was reported in the previous studies [4, 12, 13], microarray datasets focused on lung cancer was used to detect AA genes, which were verified in other tumors.

## Methods

### Gene expression microarray datasets

Five gene expression datasets introduced in this study were from ArrayExpress (http://www.ebi.ac.uk/arrayexpress/) of the European Institute of Bioinformatics (EBI). The analyses included independent cohorts containing non-small cell lung cancer (NSCLC: E-GEOD-19188 [14] and E-GEOD-37745 [15]), cervical cancer (E-GEOD-9750 [16]), breast cancer (E-GEOD-21422 [17]), hepatocellular carcinoma (E-GEOD-14520 [18]), all of which were publicly available. These datasets employed Affymetrix GeneChip Human Genome U133 plus 2, U133A, U133A_2 or HT_HG-U133A. The CEL files containing the raw data from each experiment were directly downloaded from the EBI with particular accession number. Besides, another dataset from GDC Data Portal (https://gdc-portal.nci.nih.gov/projects/TCGA-LUAD) contained normalized RNAseq data (RSEM: RNA-Seq by Expectation Maximization) of lung adenocarcinoma based on IlluminaHiSeq was also downloaded. Except for E-GEOD-37745, each dataset included the case group and the corresponding control group (Table 1).

**Table 1:** Six independent datasets from ArrayExpress and GDS website. Except for the RNAseq data (normalized RSEM expression), other gene expression microarray datasets were normalized using RMA with R-package “affy”

Cancer	Accession Number	Array	Sample Size	
			Control	Size
Non-small-cell lung cancer	E-GEOD-19188	HG-U133_Plus_2	65	91
	E-GEOD-37745	HG-U133_Plus_2	-	196
Lung Adenocarcinoma	TCGA-LUAD	IlluminaHiSeq	515	59
Cervical Cancer	E-GEOD-9750	HG-U133A	24	33
Breast Cancer	E-GEOD-21422	HG-U133_Plus_2	5	14
Hepatocellular Carcinoma	E-GEOD-14520	HT_HG-U133A	212	222

### Identification of differentially expressed genes

Raw data retrieved from ArrayExpress were then normalized using Robust Multi-array Analysis (RMA) with “affy” R-Package (version 3.2.2), and the normalized expression values represented the probe set intensity on a log-2 scale. The expression levels of more than two probes standing for the same gene were averaged. Moderated *t*-statistic was carried out with “limma” R-Package to indentify differentially expressed genes (DEGs) between different disease statuses in each dataset. Both adjusted *P* value (Benjamini & Hochberg, *P*_a_) and Fold Change (FC) were obtained and only the genes with *P*_a_ value < 0.05 and |FC| > 1.5 were selected as DEGs.

### Construction of gene co-expression network with AA genes

Pearson’s correlation coefficient (*r*) was calculated. If *P* values of correlation coefficients between ALDOA and other DEGs in the cancer group were larger than 0.05, these DEGs were regarded as AA genes. Then, if *r* value between each two AA genes was larger than 0.7 in the cancer group, these two genes were connected and considered for ALDOA-associated gene co-expression network (GCN) construction. GCN is undirected with each node corresponding to one gene. Given a significant co-expression relationship exists between a pair of nodes, they are connected with an edge. GCN is of biological interest since co-expressed genes are likely controlled by the same transcriptional regulatory program, functionally related, or members of the same pathway [19]. Besides, network analysis was performed on GCN in Cytoscape (version 3.2.1). The genes with highest degree and betweenness were considered as hub genes. Degree is defined as the number of links incident upon a node. Betweenness, an indicator of a node's centrality in a network, is equal to the number of shortest paths from all vertices to all others that pass through that node. A node with high betweenness has a large influence on the transfer of items through the network [20].

### Partial correlation analyses between ALDOA and other genes

Given the possibility that the concurrence of up-regulated ALDOA and AA genes could be a mutual effect of accelerated glycolysis in cancer, we revaluated their relationship by partial correlation analysis, which measures the degree of association between two random variables in statistics, removing the possible influences of glycolysis on the co-existence of ALDOA and AA genes.

As reported, there are three important rate limiting enzymes, namely hexokinase, phosphofructokinase and pyruvate kinase, controlling the flux of glycolysis. Hexokinase (HK) is an uninversally expressed enzyme catalyzing the conversion of glucose into glucose-6-phosphate. Phosphofructokinase (PFK) is responsible for the phosphorylation of fructose-6-phosphate yielding fructose-1,6-bisphosphate. Pyruvate kinase (PK) catalyzes the last step of glycolysis transferring a phosphate group from phosphoenolpyruvate to ADP, producing one molecule of pyruvate and one molecule of ATP. HK2, PFKM and PKM encode the muscle-type isozymes respectively of these three enzymes. Here, the partial correlation coefficients (*r*_p_) between ALDOA and other genes at the transcriptional level were calculated using R-package ‘corpcor’ when separately removing the effect of these genes.

### Network clustering and identification of cluster function

ClusterOne plugin in Cytoscape is designed to discover densely connected and possibly overlapping regions within the Cytoscape network (eg., Protein-Protein interaction network) by a greedy procedure adding or removing vertices to find groups with high cohesiveness [21]. In this study, we utilized ClusterOne to detect highly interconnected region (Cluster) of the ALDOA-associated GCN. The functional enrichment analysis was performed using DEVID bioinformatics resource (version 6.7), to explore the well known database: Gene Ontology (GO) database. We especially annotated the clusters with GO BP (Biological Process), MF (Molecular Function), and CC (Cellular Component) terms. For multiple hypothesis tests, *P*_a_ was obtained with bonferroni method.

### Patients and tissue homogenate preparation

Frozen breast tumors stored in RNAlater® Solution (P/N: AM7021, Ambion, USA) were obtained from 16 patients diagnosed and operated in the Cancer Hospital of Shantou University Medical College in 2015. All patients did not receive radiotherapy or chemotherapy before surgery resection. Informed consent for the use of their samples was obtained from all the patients. This study was approved by the medical ethics committee of the Cancer Hospital of Shantou University Medical College. Tissue samples about 500 mg were homogenized in 1 ml TRIzol (Cat No. 15596–026, Invitrogen, USA) using a tissue homogenizer, and then supernatant was extracted for mRNA quantification after centrifugation.

### Quantitative real-time polymerase chain reaction (qRT-PCR)

Transcripts of ALDOA and several hub genes were measured in breast tumors by qRT-PCR. Total-RNA was extracted from tissue homogenizer, and then reversely transcribed to cDNA was using PrimeScript™ RT reagent Kit with gDNA Eraser (Code No. RR047A, Takara, Japan). Subsequently, the expression was measured by qRT-PCR in SYBR® Premix Ex Taq™ II (Tli RNaseH Plus) (Code No. RR820A, Takara, Japan), using the gene-specific primers (Table 2). The parameters for PCR amplification were 95 °C for 2 min, followed by 40 cycles of 95 °C for 15 s and 60 °C for 1 min, 40 cycles. β-Actin was selected as the internal control. The relative mRNA expression was calculated with the comparative △Ct method using the formula 2^-△△Ct^ [22].

**Table 2:** Primers of ALDOA, CDC20, CDC45 and CCNB2 for qRT-PCR. Primer Premier 5.0 was used to design the primers, and Primer-BLAST tool of NCBI was used to ensure the accuracy and specificity of these primers

Targeted Gene	Primer	Sequence (5′–3′)
ALDOA	Forward	ATGCCCTACCAATATCCAGC
	Reverse	GACAGCCCATCCAACCCT
CDC20	Forward	GGCACCAGTGATCGACACATTCGCAT
	Reverse	GCCATAGCCTCAGGGTCTCATCTGCT
CCNB2	Forward	GCGTTGGCATTATGGATCG
	Reverse	TCTTCCGGGAAACTGGCTG
CDC45	Forward	TGGACTGCACACGGATCT
	Reverse	AACCTGGCTGCGGTATAG
TK1	Forward	TGGCTGTCATAGGCATCGAC
	Reverse	CCAGTGCAGCCACAATTACG
β-Actin	Forward	AGCGAGCATCCCCCAAAGTT
	Reverse	GGGCACGAAGGCTCATCATT

### Relationship of ALDOA with several hub genes

Spearman’s rank correlation coefficients (*r*_s_) were calculated between ALDOA and each of hub genes at the transcriptional level obtained from qRT-PCR mentioned above. *P* < 0.05 was considered as statistical significance.

### Cell lines and culture conditions

One human breast cancer cell line SKBR3 were purchased from the Culture Collection of the Chinese Academy of Sciences, Shanghai, and maintained in DMEM (high glucose) (Gibco, Thermo Fisher Scientific Inc., California, USA) supplemented with 10% fetal bovine serum (FBS, Biological Industry, Kibbutz Beit Haemek, Israel) at a 37 °C, 5% CO_2_ incubator.

### Western blotting (WB)

Cells were lysed with a lysis buffer and PMSF (Beyotime, Shanghai, China) on ice for 30 min and centrifuged at 12000 rpm for 15 min at 4 °C. Cell lysates (50 μg) were electrophoresed on 12% SDS polyacrylamide gel and transferred onto a PVDF membrane. After blocking with Tris buffered saline containing 0.05% Tween 20 (TBST) and 5% non-fat milk for 1 h at room temperature, the filters were washed 3 times × 5 min with TBST and then incubated with either rabbit anti-ALDOA polyclonal antibody (1:2000, Code No. ab71433, abcam, Cambridge, UK), or mouse anti β-Tubulin monoclonal antibody (1:1000, Code No. HC101-01, transgene, Illkirch Graffenstaden Cedex, France) diluted in blocking buffer at 4 °C overnight. After 3 times× 5 min wash with TBST, the membranes were incubated with horseradish peroxidase-labelled antirabbit (1:5000, Novus Biologicals, Littleton, USA) or antimouse (1:5000, Santa Cruz Biotechnology, Santa Cruz, USA) IgG at room temperature for 2 h, and washed with TBST. The blots were visualized with chemiluminescence.

### Knockdown of ALDOA expression by small interfering RNA (siRNA)

SKBR3 with abundant ALDOA expression, was used to test the effect of ALDOA on cell cycle progression. We tried four different pairs of siRNAs designed targeting ALDOA specifically and purchased from GenePharmagps (Shanghai, China), only one of them substantially reduced the expression level of ALDOA. Therefore we used this siRNA oligonucleotide targeting the coding sequence of human ALDOA mRNA (siALDOA) and one scramble control siRNA (siNC) was served as control. The sense and antisense strand sequences of siALDOA are 5′-GCCUUGCCUGUCAAGGAAATT−3′ and 5′-UUUCCUUGACAGGCAAGGCTT−3′, respectively, the sense and antisense strand sequences of siNC are 5′-UUCUCCGAACGUGUCACGUTT-3′ and 5′- ACGUGACACGUUCGGAGAATT−3′, respectively. When cells growing in DMEM (high glucose) supplemented with 10% FBS were at a 40–50% confluence, siALDOA or siNC was added for transfection at a ratio of 75pmol siRNA: 7.5uL Lipofectamine 3000TM (Invitrogen, Thermo Fisher Scientific Inc., California, USA), 48 h after transfection, the transfectants were either lysed to check the efficiency of knockdown by WB, or switched to glucose-free DMEM for additional 8 h culture.

### Flow cytometry for cell cycle analysis

Considered that the role of ALDOA in cancer could be an epiphenomenon of glycolysis (Warburg effect), we performed flow cytometry assays to examine the effect of ALDOA on cell cycle progression in the absence of glucose. Briefly, 48 h after transfection with either siALDOA or siNC, SKBR3 cells were then cultured in glucose-free DMEM (Gibco, Thermo Fisher Scientific Inc., California, USA) for additional 8 h to block the initiation of glycolysis, and cells were then collected and fixed with 70% ethanol at 4 °C overnight, then washed twice with ice-cold PBS. After that, 1 mg/ml RNaseA (Sigma-Aldrich Co., St Louis, MO, USA) was added at 37 °C, followed by propidium iodide (PI) staining for 30 min in the dark. BD Accuri^TM^ C6 flow cytometer was used to measure the DNA contents. Each experiment was repeated at least three times.

### Hierarchical clustering and survival analysis

Hierarchical clustering was performed to cluster samples with hub genes plus ALDOA, in order to determine whether these genes could also distinguish tumors from controls for other cancer types.

**Fig. 1:**
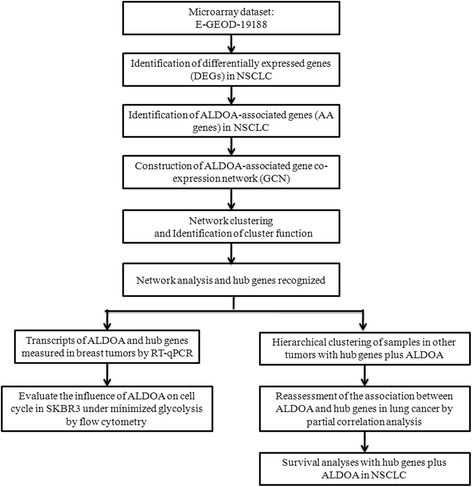
Study flow chart of this study

For the dataset E-GEOD-37745 including 196 NSCLC patients, gene expression higher or lower than median was placed in “high” or “low”. Kaplan-Meier survival analysis was conducted. The 3-year and 5-year overall survival (OS) rates were compared by *Z*-test. All analyses were carried out using the open source statistical tool R (version 3.2.2). A flow diagram depicting the whole data process in this paper was showed in Fig. 1.

## Results

### DEGs were identified between NSCLC and controls

The dataset E-GEOD-19188 based on the HG-U133_Plus_2 array, available from ArrayExpress database, contained 91 NSCLC tumors and 65 normal lung tissues (Table 1). Linear models identified 3448 genes differently expressed between NSCLC tumors and normal lung tissues (|FC| > 1.5 and *P*_a_ < 0.05), which were regarded as DEGs. Of these, 1479 genes including ALDOA were up-regulated in tumors and the remaining 1969 genes were down-regulated.

### AA genes were recognized in NSCLC

To recognize DEGs whose expression in the tumors were associated with ALDOA expression (AA genes), Pearson’s correlation coefficient was conducted within the NSCLC cancer cohort (91 arrays). Consequently, 1200 DEGs with *P* < 0.05 were identified as AA genes, of which the expression of 710 genes was positively correlated with ALDOA expression (the range of *r* values: 0.21 to 0.61), while 490 genes were negatively correlated with ALDOA (the range of *r* values:−0.54 to−0.21). Previous studies suggested genes with positively correlated expression profiles were much more likely to share similar functional annotations than genes with negatively correlated expression profiles [23]. Therefore, only these 710 AA genes were used for ALDOA-associated GCN construction.

### ALDOA-associated GCN was constructed in NSCLC

In order to construct ALDOA-associated GCN, for each pair of AA genes, we calculated Pearson’s correlation coefficients over again between their mRNA expression profiles. Totally, 1619 gene pairs with *r* ≥ 0.7 turned out to be interrelated and connected in the network. Thus the ALDOA-associated GCN finally included 182 nodes and 1619 edges.

### ALDOA-associated GCN was clustered and annotated

In our study, ClusterOne was used to identify clusters from ALDOA-associated GCN and the minimum size of cluster was set to 3 and minimum density was 0.5. Finally, 11 clusters with *P* <0.05 were detected. Cluster 1 was the biggest cluster with 79 AA genes, then followed by Cluster 2 with 22 AA genes and Cluster 3 with 5 AA genes. In view of only the first three clusters with size larger than 3 nodes, DAVID for functional enrichment analysis was performed for these clusters, and all of them were significantly enriched. The representative GO terms were listed in Table 3. Over half of genes (43/79, 54.4%) in Cluster 1 primarily involved in BP, including cell cycle process (*P*_a_ = 6.76E-26), mitotic cell cycle (*P*_a_ = 4.09E-19), DNA repair (*P*_a_ = 1.13E-04), M phase of meiotic cell cycle (*P*_a_ =0.006), positive regulation of ubiquitin-protein ligase activity during mitotic cell cycle (*P*_a_ = 0.014). ATP binding (*P*_a_ = 3.86E-05) was the main MF of Cluster 1, while nuclear lumen (*P*_a_ = 1.20E-08) and microtubule cytoskeleton (*P*_a_ = 1.60E-09) were the primary CC. Distant from Cluster 1, Cluster 2 showed MF of structural constituent of cytoskeleton (*P*_a_ =0.008) and structural molecule activity (*P*_a_ =0.009), and CC of desmosome (*P*_a_ =0.018). The only MF category in Cluster 3 was calcium ion binding (*P*_a_ =4.99E-04). Given the presentation of previous study that there was a relationship between carbohydrate metabolism and cell cycle regulation, we further focused on the genes in Cluster 1 [10].

**Table 3:** Functional enrichment of AA genes in clusters. Functional enrichment analysis was performed for cluster annotation using DEVID bioinformatics resource, exploring the well known database: Gene Ontology (GO) database. We especially annotated the clusters with GO BP (Biological Process), MF (Molecular Function) and CC (Cellular Component) terms

#Cluster	Gene size	GO	Term ID	Description	Count	*P*_a_ values*
1	79	BP	GO:0022402	cell cycle process	35	6.76E-26
			GO:0000278	mitotic cell cycle	26	4.09E-19
			GO:0006281	DNA repair	12	1.13E-04
			GO:0051327	M phase of meiotic cell cycle	7	0.006
			GO:0051437	positive regulation of ubiquitin-protein ligase activity during mitotic cell cycle	6	0.014
		MF	GO:0005524	ATP binding	22	3.86E-05
		CC	GO:0031981	nuclear lumen	27	1.20E-08
			GO:0015630	microtubule cytoskeleton	19	1.60E-09
2	22	MF	GO:0005200	structural constituent of cytoskeleton	4	0.008
			GO:0005198	structural molecule activity	7	0.009
		CC	GO:0030057	desmosome	3	0.018
3	5	MF	GO:0005509	calcium ion binding	5	4.99E-04

*P value was adjusted by Bonferroni method (*P*_a_)

**Fig. 2:**
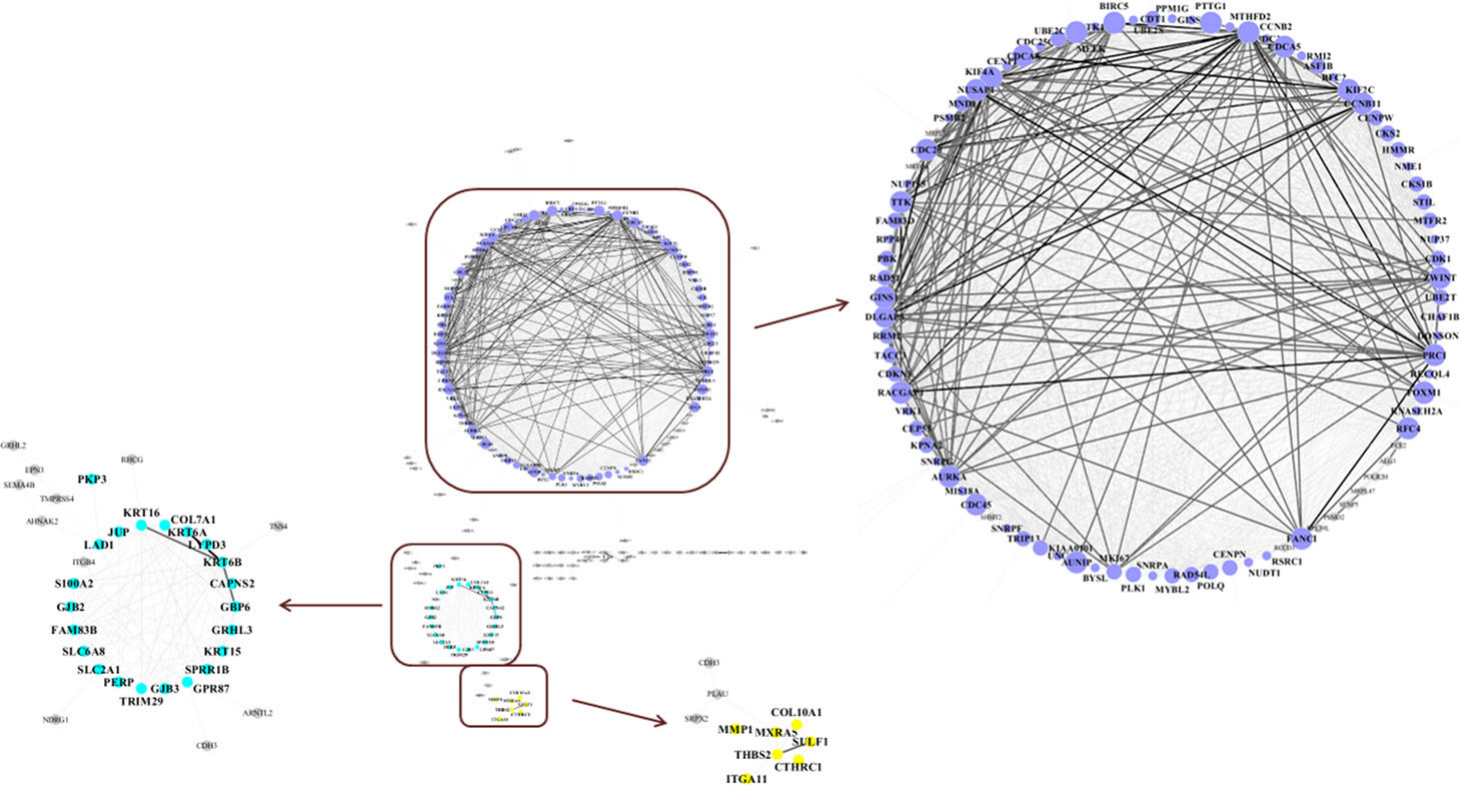
ALDOA-associated gene co-expression network (GCN). Different degree of nodes were represented by distinct sizes, and correlation coefficients between AA genes were represented by distinct color of edge. Sizes of nodes were increasing with the degree of nodes, while the color of edge was darker with ascending correlation coefficients. Purple nodes were the genes in Cluster 1, while blue nodes were the genes in Cluster 2, and yellow nodes were in Cluster 3. Genes out of these clusters were stained in grey. Cluster 1, 2 and 3 in the red rectangle were enlarged to be distinct

GCN was subjected to network analysis using Cytoscape, and displayed in Fig. 2. Different degree of nodes was represented with distant color and size, while the color of edge indicated varied correlation coefficients between these AA genes. As shown in Fig. 2, nodes with higher values of degree and betweenness were primarily centralized in Cluster 1, of which the top ranked 10 AA genes were listed in Table 4. CDC20, MELK, PTTG1, CCNB2, CDC45, CCNB1, TK1 and PSMB2 were considered as the hub genes, since these genes owned both highest values of degree and betweenness in ALDOA-associated GCN. These genes encode proteins involved in cell cycle related activities, such as cell cycle control, APC/C (Anaphase-Promoting Complex) activity, DNA replication and DNA repair, ATP catabolic process and G1/S transition.

**Table 4:** Top ranked 10 genes with highest values of either degree or betweenness. Network analysis in Cytoscape was carried out on GCN. Moderated *t*-test was performed for examining differential expression of these genes between NSCLC cases and controls (E-GEOD-19188). Pearson’s correlation coefficients were calculated between ALDOA and these genes

Gene ID	Symbol^e^	*r* ^a^	*P*_a_ value^b^	logFC^c^	*P*_a_ value^d^
991	***CDC20***	0.399	0.003	3.234	1.49E-33
9833	***MELK***	0.358	0.008	2.866	3.14E-32
9232	***PTTG1***	0.356	0.009	1.989	7.06E-26
9133	***CCNB2***	0.350	0.010	2.935	3.63E-33
8318	***CDC45***	0.414	0.002	1.933	3.09E-27
79023	**NUP37**	0.401	0.003	0.894	7.08E-20
891	***CCNB1***	0.395	0.004	2.734	1.14E-32
7083	***TK1***	0.503	<0.001	1.746	4.55E-26
5690	***PSMB2***	0.456	0.001	0.748	1.39E-19
29089	**UBE2T**	0.363	0.007	2.868	2.27E-38
55143	*CDCA8*	0.351	0.010	1.817	8.39E-32
55165	*CEP55*	0.372	0.006	2.754	5.51E-30

^a^ *r* was the Pearson’s correlation coefficient of this gene with ALDOA; ^b^ *P*_a_ value was calculated by the Pearson’s correlation method adjusted by Benjamini & Hochberg method.; ^c^logFC represented log base 2 of FC, which was the expression level ratio between cancer and control; ^d^ *P*_a_ value was calculated by the Moderated t-statistic method adjusted by Benjamini & Hochberg method. ^e^The bond symbols represented top ranked genes with highest values of degree, while the italic symbols indicated top ranked genes with highest values of betweenness

### AA genes involved in cell cycle could be novel gene signatures for lung cancer and other solid tumors

**Fig. 3:**
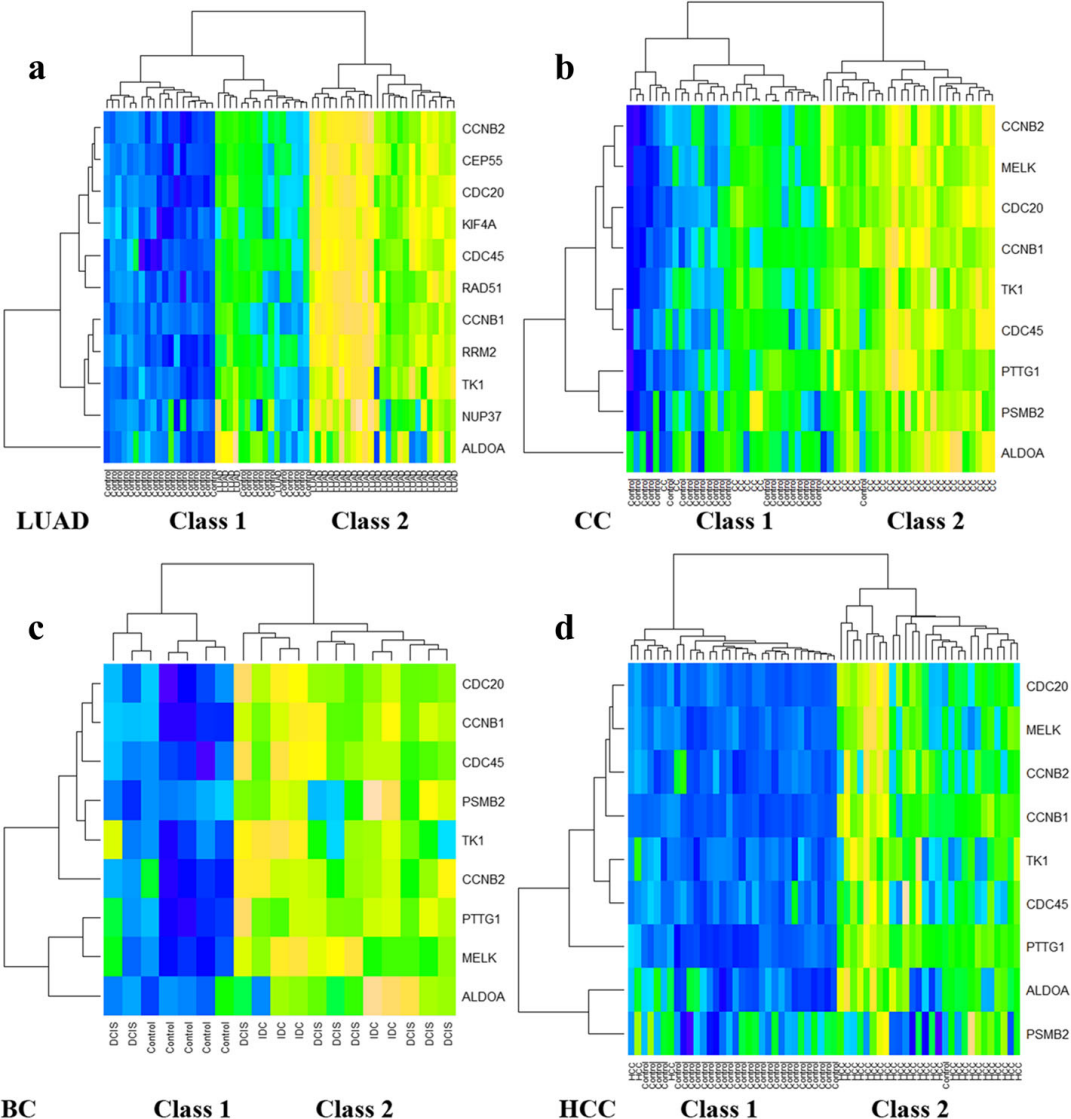
Hierarchical clustering of the hub genes and ALDOA in various cancer cohorts. Each row of a microarray heat map represented one of the AA genes with each column representing a different sample. The gene expression values from four cohorts of (**a**) lung adenocarcinoma (LUAD) with different disease states (case or control), (**b**) cervical cancer (CC) with disease states (case or control), (**c**) breast cancer (BC) with different disease states (IDC, DCIS or control), and (**d**) hepatocellular carcinomas (HCC) with different disease states (case or control) were clustered and presented by heat map

Using various datasets available to us, we evaluated whether hub genes in Cluster 1 involved in cell cycle can distinguish cancer from normal controls for lung cancer and other solid tumors. As shown in Fig. 3, elevated expression of hub genes plus ALDOA were primarily clustered in the tumors.

The cohort from TCGA-LUAD, which included 30 LUAD patients and 30 normal controls randomly sampled from the whole dataset were clustered according to expression of hub genes and ALDOA. Class 1 was associated with controls (30/35, 85.7%), while Class 2 was preferentially enriched for LUAD (25/25, 100%, Fig. 3a). Only 5 LUAD cases (5/60, 8.3%) were misclassified into Class 1.

The cohort from E-GEOD-9750 containing 33 cervical cancer patients and 24 normal controls (normal cervix epithelium), were clustered. Class 1 was mainly enriched for controls, as it has 23/29 normal controls (79.3%) and 6 cervical cancer patients. On the other hand, Class 2 centralized 27 patients with cervical cancers and 1 normal controls (Fig. 3b). Totally, 7 samples (7/57, 12.3%) were wrongly classed into the opposite.

The cohort from E-GEOD-21422, which includes 14 breast cancer patients (5 with invasive ductal breast cancer (IDC) and 9 with ductal carcinoma in situ (DCIS)) and 5 normal controls, were clustered. Class 1 had 5 normal controls (71.4%) and 2 DCIS, while Class 2 had 7 DCIS and 5 IDC (100%, Fig. 3c).

The cohort from E-GEOD-14520 containing 30 hepatocellular carcinoma patients and 30 normal controls (liver non-tumor tissue) randomly selected from the original dataset were clustered. Class 1 was preferentially associated with normal controls (29/32, 90.6%). On the other hand, Class 2 was enriched for hepatocellular carcinoma patients, as it was composed of 27 cases (27/28, 96.4%) and 1 normal controls (Fig. 3d).

### Positive relationship of ALDOA with hub genes is not regulated by glycolysis

Since HK2, PFKM and PKM2 encode the rate limiting enzymes of glycolysis. Herein, we firstly examined the relationship of ALDOA transcripts with that of these three genes, to look for indirectly the role of ALDOA in aerobic glycolysis (Warburg effect). In the dataset GSE19188, ALDOA was significantly associated with HK2 (*r* = 0.371, *P* = 2.96E-04) and PKM (*r* = 0.406, *P* = 6.39E-05), but not with PFKM (*r* =−0.057, *P* = 0.590). Likewise in the dataset TCGA-LUAD, ALDOA was also positively associated with HK2 (*r*_s_ = 0.177, *P* = 5.27E-05) and PKM (*r*_s_ = 0.583, *P* < 2.20E-16). In addition, it is significantly correlated to PFKM (*r*_s_ = 0.088, *P* = 0.046). Thus, a consistently positive relationship between ALDOA and HK2/PKM was both observed in these two datasets. Considered that HK2 as well as PKM acted as key regulators of glycolysis, these two genes could be confounders in assessing the role of ALDOA in regulating cell cycle. Therefore, secondly, we reassessed the relationship of ALDOA and hub genes with highest network degree/betweenness at the transcriptional level while excluding the possible effect of HK2 or PFKM by partial correlation analyses. As shown in Table 5, the positive relationship of ALDOA with most hub genes remained to be observed under conditions controlling the expression of HK2 or PKM. Notably and interestingly, a steady close association between TK1 and ALDOA was observed in both these two datasets.

**Table 5:** Partial correlation analyses for reassessing the relationship of ALDOA and hub genes. Partial correlation analyses were performed in R-package ‘corpcor’ to reassess the relationship between ALDOA and hub genes (the top ranked 10 genes with highest values of either degree or betweenness)

	Symbol	HK2		PKM	
		*r*_p_^a^	*P* ^b^	*r*_p_^c^	*P* ^d^
GSE19188	**TK1**	0.408	6.45E-05	0.424	3.14E-05
	CDC45	0.302	3.82E-03	0.356	5.78E-04
	CCNB1	0.290	5.51E-03	0.364	4.19E-04
	CDC20	0.284	6.77E-03	0.336	1.19E-03
	NUP37	0.275	8.74E-03	0.370	3.28E-04
	UBE2T	0.264	1.20E-02	0.326	1.73E-03
	CDCA8	0.254	1.60E-02	0.278	7.87E-03
	CEP55	0.236	2.50E-02	0.310	2.93E-03
	CCNB2	0.230	2.90E-02	0.323	1.87E-03
	MELK	0.220	3.70E-02	0.299	4.18E-03
	PSMB2	0.206	5.20E-02	0.163	1.25E-01
	PTTG1	0.193	6.90E-02	0.369	3.40E-04
TCGA-LUAD	**TK1**	0.442	<2.2E-16	0.296	7.44E-12
	PSMB2	0.438	<2.2E-16	0.334	7.99E-15
	UBE2T	0.384	<2.2E-16	0.318	1.44E-13
	CDC20	0.369	<2.2E-16	0.271	4.34E-10
	CCNB1	0.367	<2.2E-16	0.242	2.76E-08
	PTTG1	0.347	4.44E-16	0.289	2.39E-11
	NUP37	0.346	6.66E-16	0.190	1.44E-05
	CCNB2	0.312	4.59E-13	0.150	6.60E-04
	CDC45	0.297	6.85E-12	0.184	2.77E-05
	CDCA8	0.287	3.08E-11	0.189	1.60E-05
	CEP55	0.263	1.40E-09	0.143	1.18E-03
	MELK	0.242	2.80E-08	0.157	3.64E-04

^a^ *r*_p_ was the correlation coefficient between ALDOA and the other genes using partial correlation analyses with *P* values (^b^ *P*) when controlling HK2 expression at the transcriptional level; Likewise, ^c^ *r*_p_ was the partial correlation coefficient between ALDOA and the other genes with *P* values (^d^ *P*) when controlling PKM expression at the transcriptional level

### Relationship of ALDOA with several hub genes were verified in breast tumors

**Fig. 4:**
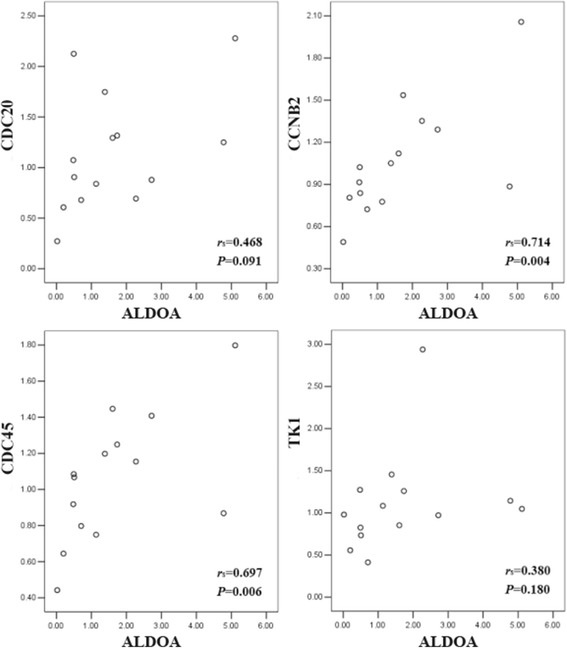
mRNA levels of ALDOA detected by RT-qPCR and its relationship with CDC20, CDC45, CCNB1 and TK1. The horizontal axis and vertical axis of each scatter plot represented relative transcriptional expression of each gene. *P* values were received from Spearman’s correlation analysis

Given that ALDOA and hub genes could identify most cancers from normal controls using public datasets which were demonstrated above, we further examined the mRNA levels of ALDOA and several hub genes in breast tumors by RT-qPCR. 16 independent tumors were included. However, only transcripts of 14 patients were put into analysis for 2 tumors with detection error and inconsistence among three replicates. Transcripts of four hub genes (CDC20, TK1, CCNB2 and CDC45) and ALDOA were detected and Spearman Correlation Coefficients were calculated. As shown in Fig. 4, the correlation of ALDOA with two hub genes were confirmed. The *r*_s_ between CCNB2 and ALDOA was 0.714 (*P* = 0.004), while *r*_s_ between CDC45 and ALDOA was 0.697 (*P* = 0.006). Although the positive trend between ALDOA and CDC20 or TK1 was observed, the correlations between them (*r*_s_ = 0.468, *P* = 0.091; *r*_s_ = 0.380, *P* = 0.180) were failed to meet statistical significance.

### ALDOA might affect the cell cycle progression independent of glycolysis

**Fig. 5:**
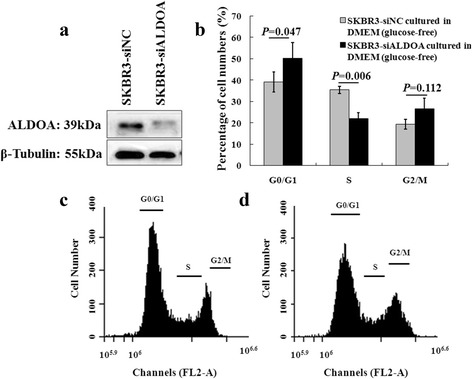
Evaluation of ALDOA influence on cell cycle in SKBR3 under minimized glycolysis by flow cytometry. (**a)** WB indicated an obviously downregulated ALDOA expression in SKBR3-siALDOA compared to that in SKBR3-siNC. (**b**) Knockdown of ALDOA significantly increased the percentage of cells in G0/G1 phase (39.15 ± 4.75% vs. 50.32 ± 7.44%, *P* = 0.047), accompanied by a considerable decrease in the percentage of cells in S phase (35.41 ± 1.71% vs. 21.95 ± 2.80%, *P* = 0.006). No significant changes were seen in the percentage of cells in G2/M phase (19.40 ± 2.27% vs. 26.54 ± 5.15%, *P* = 0.112). (**c**) and (**d**), representative DNA histograms of SKBR3 transfected with siNC (SKBR3-siNC, C) or siALDOA (SKBR3-siALDOA, D) after incubation in glucose-free median for additional 8 h

According to previous reports [24], 8 h of incubation in glucose-free medium leaded to rapid reduction of the ATP levels, an indicator of glycolysis, in breast cancer cells. Therefore, 48 h after transfection, we switched SKBR3 cells transfected with either siALDOA or siNC to glucose-free DMEM for additional 8 h to minimize the effects of glucolysis. Then proceeded to flow cytometry analysis. Shown in Fig. 5 was the average of three independent experiments, compared to SKBR3-siNC, knockdown of ALDOA significantly increased the percentage of cells in G0/G1 phase (39.15 ± 4.75% vs. 50.32 ± 7.44%, *P* = 0.047), which was accompanied by a considerable decrease in the percentage of cells in S phase (35.41 ± 1.71% vs. 21.95 ± 2.80%, *P* = 0.006). There were no significant changes in the percentage of cells in G2/M phase (19.40 ± 2.27% vs. 26.54 ± 5.15%, *P* = 0.112).

### ALDOA might improve the predictive power of hub genes for NSCLC

**Fig. 6:**
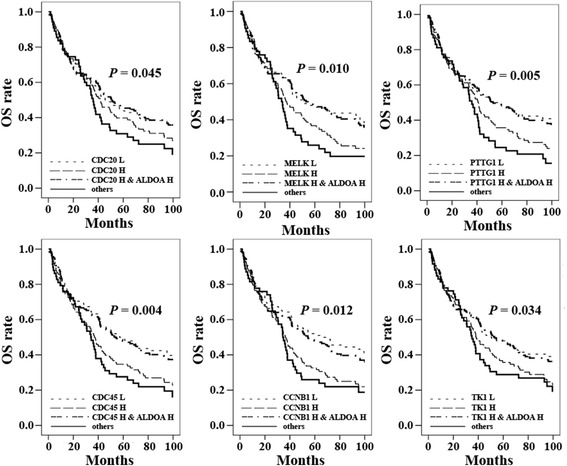
Prognostic significance of up-regulation of hub genes and ALDOA in lung adenocarcinoma. Kaplan-Meier survival analysis was performed on this cohort from E-GEOD-37745 including 196 patients with lung adenocarcinoma. Up-regulation of these selected AA genes were associated with poor prognosis. Combination of elevated ALDOA expression with individual AA gene improved the prediction power. *P* values were obtained from Kaplan Meier analysis comparing survival rates between patients with high expression of both ALDOA and each hub gene, and patients with expression of other levels. H = high. L = low

To evaluate whether hub genes with or without ALDOA could be biomarkers to predict cancer prognosis, survival analyses were conducted using the dataset E-GEOD-37745, which composed of survival time and outcomes of 196 NSCLC patients. As shown in Fig. 6, high expression of CDC20 had a trend to be associated with poor outcome, and ALDOA could improve the predictive power (log-rank *P* = 0.045). Similar results were observed of MELK (*P* = 0.010), PTTG1 (*P* = 0.005), CDC45 (*P* = 0.004), CCNB1 (*P* = 0.012) and TK1 (*P* = 0.034). Besides, both 3-year and 5-year OS rates of different hub gene levels were shown in Table 6, indicating that patients with high expression of ALDOA plus hub genes indicated worse survival rates, especially the 5-year OS rate.

**Table 6:** 3-year and 5-year OS rates of patients with different expression level of AA genes with or without ALDOA expression. *Z*-test was used to compare OS rates between different groups

AA genes^a^	Sample size	3-year OS			5-year OS		
		OS rate	*Z* ^b^	*P*	OS rate	*Z* ^b^	*P*
CDC20 L	98	0.582	0.156	0.876	0.439	0.582	0.561
CDC20 H	98	0.571			0.398		
CDC20 H ALDOA H	55	0.491	1.515	0.130	0.309	1.938	0.053
others	141	0.61			0.461		
MELK L	98	0.52	1.601	0.109	0.367	1.447	0.148
MELK H	98	0.633			0.469		
MELK H ALDOA H	54	0.426	2.947	0.003	0.259	3.122	0.002
others	142	0.634			0.479		
PTTG1 L	98	0.592	0.439	0.661	0.480	1.745	0.081
PTTG1 H	98	0.561			0.357		
PTTG1 H ALDOA H	53	0.491	1.472	0.141	0.245	3.000	0.003
others	143	0.608			0.483		
CDC45 L	98	0.633	1.601	0.109	0.490	2.029	0.042
CDC45 H	98	0.52			0.347		
CDC45 H ALDOA H	58	0.466	2.031	0.042	0.276	2.617	0.009
others	138	0.623			0.478		
CCNB1 L	98	0.643	1.884	0.060	0.510	2.597	0.009
CCNB1 H	98	0.51			0.327		
CCNB1 H ALDOA H	54	0.463	1.987	0.047	0.259	2.790	0.005
others	142	0.62			0.479		
TK1 L	98	0.612	1.005	0.314	0.480	1.745	0.081
TK1 H	98	0.541			0.357		
TK1 H ALDOA H	59	0.475	1.885	0.059	0.288	2.421	0.015
others	137	0.62			0.439		

^a^Patients in the cohort E-GEOD-37745 were divided into one group of patients with low expression of hub gene and the other group of patients with high expression of hub gene, or divided into one group of patients with high expression of both hub gene and ALDOA, and the other group of patients with other expression levels; ^b^The difference of 3-year and 5-year OS rates between the group of patients with low and high expression of hub gene, or between the group of patients with high expression of both AA and ALDOA, and the other group of patients with other expression levels were compared by *Z*-test respectively

## Discussion

In this study, we utilized the available microarray datasets from the public database, ArrayExpress and GDC, to evaluate transcriptional levels of ALDOA and AA genes in solid tumors. The dataset used for detecting the DEGs and AA genes relied on an independent NSCLC cohort (E-GEOD-19188), but not aggregated data for avoiding false positive and/or negative correlation resulted from combining different datasets from various sources. Our identification of statistically significant changes in ALDOA expression in NSCLC enabled the evaluation of the gene expression profile that associated with ALDOA. In this study, AA genes in tumors were identified through ALDOA-associated GCN construction and clustering, to help explaining the role of ALDOA in tumors.

ALDOA has been known as the sole aldolase isozyme in red blood cells and skeletal muscle and is necessary for the production of adenosine triphosphate (ATP) in erythrocytes and muscle fibers [3]. Recently, increasing evidences have shown that ALDOA could express in cancer cells, and its contribution to carcinogenesis in some tumors has been proposed both in vivo and in vitro. The role of ALDOA in lung cancer has been widely studied. Rho et al. [13] has pointed that ALDOA protein was up-regulated in human lung adenocarcinomas compared to normal pulmonary tissue, which was consistent with the result of Lin’s paper [12]. Du et al. [4] further showed that ALDOA protein may induce epithelial-mesenchymal transition and promote cell migration in lung squamous cell carcinoma. Additionally, in previous studies, positive effect of ALDOA on initialization and progression of other cancers, such as colorectal cancer, oral tumor, osteosarcoma and hepatocellular carcinoma, had also been demonstrated [5–8]. Moreover, in vitro, ALDOA mRNA levels were down-regulated after glioma cell line SHC-44 cells treated with all-trans retinoic acid [25]. Compared with melanocytes, the mRNAs of ALDOA were highly expressed in human melanoma cell lines G361 [26]. In our paper, we also confirmed that ALDOA might contribute to tumorgenesis with aberrant mRNA levels in NSCLC. All these above have demonstrated that elevated ALDOA expression might be a potential biomarker for cancer diagnosis.

However, the mechanism of ALDOA in cancer remains unknown. Although some papers suggested that this was correlated to glycolysis, Ritterson and Tolan [10] have shown that silencing ALDOA drastically decreased the rate of cancer cell proliferation, and this did not greatly interfere with cellular energy metabolism. Although ALDOA is localized primarily in the cytoplasm, nuclear localization of ALDOA might be a common feature of proliferating cells, including cancer cells. All these indicated another potential mechanism other than glycolysis that ALDOA might involved in for carcinogensis. As a supplement to these reports, our analysis further showed that a majority of AA genes (over 50%) in the biggest cluster (Cluster 1) out of ALDOA-associated GCN were enriched for biological process relevant to cell cycle control, which demonstrated that ALDOA mRNA expression in NSCLC probably involved in cell proliferation. This result turned out to be consistent with the finding of Mamczur’s paper [9], which pointed to ALDOA as a factor involved in the regulation of cells proliferation in lung cancer cell. Mamczur and his colleagues further showed that ALDOA tended to co-existed with the expression of MKI67, a marker of proliferation. Interestingly, our paper also displayed a positive relationship between ALDOA and MKI67 (*r* = 0.275, *P*_a_ = 0.046), and MKI67 was included in Cluster 1 (Fig. 2).

Moreover, we performed network analysis of GCN, and in view of the degree and betweenness of nodes, CDC20, MELK, PTTG1, CCNB2, CDC45, CCNB1, TK1 and PSMB2 were identified as the hub genes (Table 4), all of which directly or indirectly are involved in cell cycle control. Except for PSMB2, the relationship between these hub genes and cancer has been reported by previous studies. TK1, the most ALDOA relevant gene in GCN (*r* = 0.503), is a key kinase in the one-step salvage pathway, participates in DNA synthesis and is therefore closely related to the S-phase of the cell cycle. CDC45 (*r* = 0.41) is crucial for the initiation as well as the elongation process of eukaryotic DNA replication. Both of them have been found to be upregulated in several tumors and associated with proliferating cell populations [27–29]. The APC/C’s main function is to trigger the transition from metaphase to anaphase by tagging specific proteins for degradation. CDC20 (*r* = 0.40) is a regulatory protein that activates the APC. PTTG1 (*r* = 0.36) is an APC substrate that associates with a separin until activation of the APC. Upregulation of these two genes was associated with aggressive progression and poor prognosis in several tumors [30–32]. CCNB1 [33, 34] and CCNB2 [35, 36] might contribute to G2/M transition, and function as an oncogene and serve as a potential therapeutic target. MELK is a cell cycle-dependent protein kinase that belongs to the KIN1/PAR-1/MARK family. MELK overexpression has been detected in various human tumors [37]. Given that a significant correlation between these genes and ALDOA was observed in our paper, it was supposed that ALDOA probably served as a key role in cell cycle regulation.

Our analysis also demonstrated up-regulated transcripts of ALDOA and hub genes could mostly distinguish tumors from controls not only in NSCLC, but also in other tumors, namely cervical cancer, breast cancer and hepatocellular carcinoma, suggesting that transcription of ALDOA might contribute to increased cell cycle-related cell proliferation, and be an important and probably universal step in carcinogenesis. We further detect transcripts of ALDOA and several hub genes in breast tumors by RT-qPCR, and Pearson’s correlation analysis demonstrated a positive relationship of ALDOA with CCNB2 and CDC45, but not CDC20 and TK1, even a trend observed between them. Although intimate correlation of ALDOA with several genes relevant to carbohydrate metabolism, such as LDHA (*r* = 0.605, *P*_a_ < 0.001), PFKP (*r* = 0.598, *P*_a_ < 0.001), GPI (*r* = 0.595, *P*_a_ < 0.001), TPI1 (*r* = 0.548, *P*_a_ < 0.001), GAPDH (*r* = 0.517, *P*_a_ < 0.001), PGK1 (*r* = 0.447, *P*_a_ = 0.001), PGAM1 (*r* = 0.411, *P*_a_ = 0.002), ENO2 (*r* = 0.394, *P*_a_ = 0.004), PKM (*r* = 0.363, *P*_a_ = 0.007) and ENO1 (*r* = 0.361, *P*_a_ = 0.008) was found for NSCLC, no cluster enriched into GO terms relevant to carbohydrate metabolism, indicating that ALDOA participated in carcinogenesis more likely through cell cycle control other than glycolysis. However, it might be partially due to limited number of metabolic genes found to be correlated with ALDOA, and it is unsuitable for ClusterOne used in our paper since this program tended to detect clusters with a large size of nodes.

Given that the positive relationship between ALDOA and AA genes indicated by Pearson’s correlation analysis could not exclude the possibility that it might be an entirely mutual consequence of the high energy demands required for rapid growth (Warburg effect). Thus, we reassessed their association by partial correlation analyses using GSE19188 and TCGA-LUAD by removing the potential influences of the two ALDOA-associated rate limiting enzymes of glycolysis (HK2 and PKM) [38–40]. We found that the significant association between ALDOA and most of hub genes remained, especially that between ALDOA and TK1 was seen both in these two datasets (GSE19188: *r*_p_ = 0.408/0.424; TCGA-LUAD: *r*_p_ = 0.442/0.296). Although RT-qPCR mentioned above failed to indicate a positive relationship between them (probably due to the limited sample sizes), we still supposed TK1 might be a promising target when studying the mechanism of ALDOA in cancer in future. Additionally, knocking down ALDOA in SKBR3 cells blocked cells at G0/G1 under minimized glycolytic condition, suggesting that ALDOA could contribute to the progress of cancer, at least partially through its association with genes relevant to cell cycle independent of glycolysis.

Several hub genes found in our paper have been proposed to predict poor prognosis of NSCLC in previous studies, such as CDC20 [41], MELK [42], PTTG1 [43], CCNB1 [44] and TK1 [45]. However, our study have indicated that these genes in combination with ALDOA could dramatically improve the predictive power for NSCLC prognosis. As shown in Figure 6 and Table 6, patients simultaneously with high expression of ALDOA and AA genes had a significantly lower survival rates than patients only with high expression of AA gene, especially for 5-years OS rates.

## Conclusions

Our study has displayed ALDOA mRNA upregulation in cancers, confirmed its seemingly universal effect on carcinogenesis. Positive association between ALDOA and hub genes relevant to cell cycle remained even after minimizing the effect of glycolysis, indicating that ALDOA might contribute to cell proliferation of cancer, at least partially independent of glycolysis. AA genes, especially the hub genes would help to elucidate the non-glycolytic related functions of ALDOA in cancer. ALDOA might be a potential diagnostic and prognostic factor for cancer since ALDOA could distinguish tumors from controls and dramatically improve the predictive power of AA genes for poor survival.

## Abbreviations

AA genes: ALDOA-associated genes
ALDOA: Aldolase A
BC: Breast cancer
BP: Biological Process
CC: Cellular Component
CC: Cervical cancer
DCIS: Ductal carcinoma in situ
DEGs: Differentially expressed genes
GCN: Gene co-expression network
GO: Gene Ontology
HCC: Hepatocellular carcinomas
IDC: Invasive ductal breast cancer
MF: Molecular Function
NSCLC: Non-small-cell lung cancer
*P*_a_: Adjusted *P* values
